# Alpha-lipoic acid protects against pressure overload-induced heart failure via ALDH2-dependent Nrf1-FUNDC1 signaling

**DOI:** 10.1038/s41419-020-02805-2

**Published:** 2020-07-30

**Authors:** Wenjia Li, Lei Yin, Xiaolei Sun, Jian Wu, Zhen Dong, Kai Hu, Aijun Sun, Junbo Ge

**Affiliations:** 1 Department of Cardiology, Zhongshan Hospital, Fudan University, Shanghai, China; 2 Shanghai Institute of Cardiovascular Diseases, Shanghai, China; 3 NHC Key Laboratory of Viral Heart Diseases, Shanghai, China; 4 Key Laboratory of Viral Heart Diseases, Chinese Academy of Medical Sciences, Shanghai, China; 5 Department of Urology, Shanghai Tenth People’s Hospital, Tongji University, Shanghai, China; 6 Institutes of Biomedical Sciences, Fudan University, Shanghai, China

**Keywords:** Heart failure, Experimental models of disease

## Abstract

Alpha-lipoic acid (α-LA), a well-known antioxidant, was proved to active ALDH2 in nitrate tolerance and diabetic animal model. However, the therapeutic advantage of α-LA for heart failure and related signaling pathway have not been explored. This study was designed to examine the role of α-LA–ALDH2 in heart failure injury and mitochondrial damage. ALDH2 knockout (ALDH2^−/−^) mice and primary neonatal rat cardiomyocytes (NRCMs) were subjected to assessment of myocardial function and mitochondrial autophagy. Our data demonstrated α-LA significantly reduced the degree of TAC-induced LV hypertrophy and dysfunction in wild-type mice, not in ALDH2^−/−^ mice. In molecular level, α-LA significantly restored ALDH2 activity and expression as well as increased the expression of a novel mitophagy receptor protein FUNDC1 in wild-type TAC mice. Besides, we confirmed that ALDH2 which was activated by α-LA governed the activation of Nrf1–FUNDC1 cascade. Our data suggest that α-LA played a positive role in protecting the heart against adverse effects of chronic pressure overload.

## Introduction

Heart failure is linked with high mobility and mortality rate worldwide. Structural cardiac remodeling, which includes myocardial hypertrophy and fibrosis, has been demonstrated to contribute significantly to ventricular dysfunction in heart failure^1^. Besides, abnormal mitochondrial metabolism serves as an important pathological basis of heart failure^2,3^. Understanding the role of mitochondrial metabolism in the development of heart failure is helpful for early detection of risk factors and for the application of effective therapeutic options aiming to improve the prognosis of heart failure.

Acetaldehyde dehydrogenase 2 (ALDH2) is the most active isozyme in ALDH enzyme superfamily which mainly distributes in mitochondria^4^. ALDH2 has three enzyme activities, including dehydrogenase, esterase, and reductase, and the most studied activity is the dehydrogenase activity, which can remove excess aldehyde metabolites. Our previous studies have defined its important cardio-protective role in the setting of various risk factors of heart failure, such as coronary artery disease (CAD), hypertension, diabetes, alcoholism, and other susceptibilities^5–8^. The indispensable role of ALDH2 in the pathogenesis of heart failure reveals that targeting ALDH2 might be a potential therapeutic option for heart failure and other cardiovascular diseases. Although many studies are performed to explore the efficacy of drugs capable of activating ALDH2, such as Alda-1, clinical promotion is limited due to cytotoxicity or other reasons of applied drugs^9^.

Alpha-lipoic acid (α-LA) is a coenzyme present in mitochondria, a cofactor for pyruvate dehydrogenase complex, ketoglutarate and amino acid hydrogenase complex, which belongs to a class of B vitamins. α-LA has a closed ring structure composed of sulfur carbon atoms, so its electron density is relatively high and consequently its antioxidant performance is relatively strong. It has been widely applied on diabetes patients based on its strong antioxidant properties and rare adverse reactions (incidence rate ≤1/10,000). Studies have shown that α-LA could restore the activity of ALDH2 by reducing disulfide at its active site, thereby improving nitrate tolerance^10^. Previous studies showed that α-LA could protect against diabetic cardiomyopathy and acute ischemia–reperfusion injury by restoring ALDH2 activity^11,12^. α-LA has been proved to attenuate cardiac hypertrophy in transverse aortic constriction (TAC) model^13^. However, the therapeutic advantage of α-LA for heart failure and related signaling pathway have not been explored.

Mitophagy is an evolutionarily conserved degradation pathway responsible for removing dysfunctional or superfluous mitochondria, in order to fine-tuning mitochondrial function and preserving energy metabolism^14^. FUN14 domain containing 1 (FUNDC1), is an activator of hypoxia-induced mitophagy. It binds to LC3 protein and elevates mitophagy activity which in turn prevents mitochondrial apoptosis activation, promotes mitochondrial biosynthesis and maintains ATP production^15^. It has been reported that FUNDC1 plays a significant role in cardio-protection in response to hypoxia^16^. But the interaction of ALDH2 and mitophagy in cardiac pressure-overload injure has not been fully described.

Thus, this study was designed to elucidate the role of α-LA on pressure overload myocardial injury and related mechanisms focusing on ALDH2- and FUNDC1-related mitophagy.

## Materials and methods

### Animals

All animal studies were performed according to a standard protocol approved by the Animal Care and Use Committee of Fudan University. Briefly speaking, 8-week-old male C57BL/6 wild-type (WT) mice (average weight 20–25 g) were purchased from Shanghai Animal Administration Center (Shanghai, China). C57BL/6-ALDH2-KO (ALDH2^−/−^) mice were produced by method described previously^7^. All mice were maintained in a specific pathogen free facility with 6 mice per cage at a temperature of 24 °C, with a relative humidity of 55 ± 5% and 12‐h light/12‐h dark cycles at Shanghai Zhongshan Hospital (Shanghai, China). In animal studies, both the carer of the animals and the assessor of the results are blinded.

After 1 week of acclimatization to the environment, the WT and ALDH2^−/−^ mice were randomly assigned to sham or TAC operation group. After anesthetized in an induction chamber with 2% isoflurane mixed with 0.5–1.0 L/min 100% O_2_ by inhalation, mice were placed in a supine position. The thorax was opened under sterilize conditions and the transverse aorta was dissected free at the aortic arch level of surrounding tissues and muscles. A 5-0 nylon suture was tied around the aorta and a blunted 27-gauge needle was removed after the ligation. Sham operation included all procedures except the ligation. During the surgical procedure, anesthesia is maintained at 1.5–2% isoflurane with 0.5–1.0 L/min 100% O_2_ by inhalation. All surgery procedures were performed under anesthesia using isoflurane. Immediately after TAC, the mice were randomly assigned to two groups supplied with α-LA (0.2% wt/wt)^17,18^ or vehicle (normal saline) in drinking water persistently for a total feeding period of 4 weeks^19^. At the end of the experiments, mice were euthanized by an overdose of pentobarbital (150 mg/kg, i.p). Then the hearts and lungs were harvested to measure organ weights and for histological examination or further molecule studies.

### Chemicals, reagents, and antibodies

Angiotensin II (AngII, Cat#: A9525) and Medium 199(M199, Cat#:M7528) were purchased from Sigma-Aldrich. α-Lipoic acid (α-LA, Cat#: H20059737) was purchased from Shenlong Pharmaceutical Industry.

The following antibody were used: ALDH2 (Abcam Cat# ab108306, RRID:AB_10862581), TOM20 (Cell Signaling Technology Cat# 42406, RRID:AB_2687663), LC3B (Cell Signaling Technology Cat# 3868, RRID:AB_2137707), P62 (Abcam Cat# ab56416, RRID:AB_945626), FUNDC1 (Abcam Cat# ab74834, RRID:AB_2247068), cleaved caspase-3 (Cell Signaling Technology Cat# 9664, RRID:AB_2070042), Bcl-2 (Cell Signaling Technology Cat# 3498, RRID:AB_1903907), Bax (Cell Signaling Technology Cat# 14796, RRID:AB_2716251), nuclear respiratory factor 1 (Nrf1) (Cell Signaling Technology Cat# 46743, RRID:AB_2732888), Nrf2 (Cell Signaling Technology Cat# 12721, RRID:AB_2715528), LDHA (Cell Signaling Technology Cat# 2012, RRID:AB_2137173), and PFKP (Abcam Cat# ab89003, RRID:AB_2042617). Respective proteins were analyzed with β-actin (Abcam Cat# ab179467, RRID:AB_2737344) serving as the loading control. Immunoreactivity was detected by an enhanced chemiluminescence reaction system (Amersham Pharmacia Biotech).

As for lentiviral vector construction, the pLKO/pLKO.1-sh-Nrf1 plasmids, pLKO/pLKO.1-sh-ALDH2 plasmids, pWPI/pWPI-oe-ALDH2 plasmids, pMD2G envelope plasmid, and psPAX2 packaging plasmid were transfected into HEK-293 cells using the standard calcium chloride transfection method^20^. The sequences of oligonucleotides used in this experiment were listed in Supplementary Table S3. The lentivirus soups were collected after incubating for 48 or 72 h and frozen in −80 °C for later use.

### Cells and culturing conditions

Primary neonatal rat cardiomyocytes (NRCMs) were prepared by enzymatic digestion of hearts from 1- to 3-day-old Sprague–Dawley rat pups (Male, Shanghai Animal Administration Center) as described previously^12^. NRCMs were plated onto collagen-coated culture dishes or cover slips and cultured in cardiomyocyte culture medium (M199 supplemented with 5% fetal bovine serum, 10% horse serum, 1% penicillin + streptomycin (Invitrogen, Carlsbad, CA)). To induce hypertrophy, after serum starvation for 24 h, NRCMs were cultured with 1-μM Angiotensin II (Ang II)^17,21^ for 48 h. For α-LA treatment group, NRCMs were treated with α-LA (10 μM)^22^ for 1 h before Ang II stimulation.

### Echocardiographic assessment

Four weeks post TAC surgery, echocardiography measurements were performed during mild anesthesia (1.5–2% isoflurane mixed with 0.5–1.0 L/min 100% O_2_ by inhalation). Trans-thoracic two-dimensional M-mode echocardiography was performed noninvasively using Vevo 770 imaging system (VisualSonics, Toronto, Canada) equipped with a 30 MHz highfrequency scan head. Left ventricular (LV) parasternal long-axis view during diastole and systole stage were recorded in which procedure heart rate was monitored and maintained at 450–500 beats per minute. LV ejection fraction (LVEF), LV fractional shortening (LVFS), LV end-systolic diameter (LVESD), LV end-diasolic diameter (LVEDD), and LV diastole posterior wall dimension (LVPWD) were measured as described previously^12^. All measurements were averaged of at least five consecutive cardiac cycles and were carried out by experienced technicians who were unaware of the animal groups.

### Histological staining and measurement

Hearts were arrested after anesthesia by ketamine (intraperitoneal injection, i.p. 50 mg/kg) and were rapidly fixed in 4% neutral formaldehyde or OCT compound. Heart tissues were sectioned at a thickness of 4 μm cut and stained with hematoxylin and eosin (H&E), wheat germ agglutinin (WGA), and Masson’s trichrome to assess myocardial morphology, cardiac cross-sectional area and cardiac collagen content, respectively. Frozen specimen were incubated dihydroethidium (DHE, 5 µM, Beyotime Biotechnology, China) at 37 °C for 30 min or with terminal dexynucleotidyl transferase(TdT)-mediated dUTP nick end labeling (TUNEL) kit (Roche Diagnostics, Indianapolis, IN) to assess excessive generation of myocardial reactive oxygen species (ROS) and apoptosis. All analysis were carried out in a blinded manner. Images were quantified using Image-Pro Plus software (RRID:SCR_007369).

### Cell shortening/relengthening

Using direct needle perfusion of the LV ex vivo, single mouse cardiomyocytes were isolated as described^23^. Only rod-shaped myocytes with clear edges were selected for contractile study. Mechanical properties of cardiomyocytes were assessed using a SoftEdge Myocam (IonOptix, Milton, MA). Peak shortening (PS), time-to-PS (TPS), time-to-90% relengthening (TR90), maximal velocities of shortening (+d*L*/d*t*) and relengthening (−d*L*/d*t*) were assessed during cell shortening and relengthening as described^24^.

### ALDH2 enzymatic activity and ATP measurement

The activity of ALDH2 was measured by one kind of tissue acetaldehyde dehydrogenase 2 activity colorimetric quantitative detection kit (GMS50300.2, GENMED SCIENTIFICS INC. USA) which aimed to detect the increase in the peak activity of oxidized nicotinamide adenine dinucleotide (NAD) in the reaction system using colorimetry in tissue lysate extract samples. The level of ATP (adenosine 5′-triphosphate) in tissue was detected by an ATP Assay Kit (S0026, Beyotime Biotechnology), which was based on the firefly luciferase (also known as luciferase) to catalyze the fluorescence generation of luciferin. It needs ATP to provide energy for development. When both firefly luciferase and luciferin are excessive, the production of fluorescence is proportional to the concentration of ATP in a certain concentration range. This makes it possible to detect the ATP concentration in the solution with high sensitivity. Whole cell fresh lysate from heart tissue was used to detect ALDH2 activity by monitoring NADH formation from NAD+ at 340 nm in a spectrophotometer and ATP concentration was detected by measuring RLU in a luminometer. The detection process was carried out according to the instructions of the kits.

### Transmission electron microscopy (TEM)

Freshly dissected LV myocardial tissue ≤1 mm^3^ was fixed with 2.5% glutaraldehyde (pH 7.4) for at least 2 h. After being washed in 0.1 M phosphate buffer (PB) 3 times followed by fixation with 1% osmium tetroxide, samples were dehydrated through graded alcohols and embedded in Epon Araldite. Ultrathin sections (50–60 nm) were cut using an ultrami crotome (Leika, Germany) and stained with 3% uranyl acetate and lead citrate. Images were acquired with a CM-120 transmission electron microscope (Philip, Holland).

### Mitophagy-related array and real-time PCR

The mouse mitophagy polymerase chain reaction (PCR) array were purchased from XYbiotech Company (Shanghai, China) and the detailed array contained genes information was listed in Supplementary Table S1. The primers of Anp, Bnp, β-Mhc, Ctgf, Col1a, Col3a, and β-actin used in this experiment were listed in Supplementary Table S2. Briefly, with or without 4 weeks’ α-LA treatment, myocardial tissue or cells was extracted in TRIZOL regent (Invitrogen). After RNA was separated and precipitated, a gelatinous precipitate was formed on the bottom and the side walls of the tube. Removed supernatant, and washed the RNA pellet with 75% ethanol. Dried RNA in air and used DNase I digest RNA samples to remove genomic DNA that may be contained. Performed RNA purification using RNeasy^®^ MinEluteTM Purification Kit (Qiagen). RNA yield and quality were determined by UV absorption assay (NanoDrop^®^ ND-1000) and denaturing agarose gel electrophoresis. The ratio of A260/A280 ranged from 1.8 to 2.1, and cDNA was synthesized by reverse transcription and PCR was performed by real-time PCR detection. Finally, data analysis were performed using the ΔΔCt method.

### Western blot analysis

Briefly speaking, tissue protein extraction and western blotting were carried out as described^25^. Protein mixtures was normalized to 20 µg and loaded into each well and separated by 10–15% sodium dodecyl sulfate (SDS) polyacrylamide gel electrophoresis. Following a 120 min run, the proteins were the electrophoretically transferred onto o PVDF membranes (Bio-Rad, USA). Then membranes were immersed in the blocking buffer containing 5% bovine serum albumin for 1 h prior to the incubation with primary antibodies. Horseradish peroxidase-conjugated secondary antibodies) were correspondingly used to the incubate the membranes for 2 h (Abcam, UK). Bands were visualized under the Bio-Rad chemiluminescence system (Image Lab Software, RRID:SCR_014210).

### NRCMs cell surface area

After treatment with different agents, NRCMs were rinsed and fixed with 4% paraformaldehyde for 20 min at room temperature. Next, the cells were washed and incubated with 5 µg/ml TRITC Phalloidin for 30 min at room temperature, followed by three additional washes with PBS. Subsequently, the cells were stained the nucleus using DAPI for 10 min. Following four rinses with PBS, cells were sealed and photographed with fluorescence confocal microscope (Nikon TE2000, Tokyo, Japan). Areas of NRCMs cells were measured by the NIH ImageJ software(RRID:SCR_003070).

### Luciferase reporter assay

The 2 kb promoter region of FUNDC1 was constructed into the pGL3-basic vector (Promega, Madison, WI). NRCMs were seeded in 24-well plates and were transfected with plasmids using Lipofectamine 3000 (Invitrogen) according to the manufacturer’s instruction^26^. After 48 h, cells were lysed and measured according to the manufacturer’s instruction using the Dual-Luciferase Reporter Assay System (Promega). Each sample was normalized by pRL–TK–luciferase activity and measured at least three independent experiments.

### Chromatin immunoprecipitation (ChIP) assay

ChIP experiments were performed following the Cold Spring Harbor ChIP protocol^27^. Briefly, NRCMs (2 × 10^7^) were fixed with 1% formaldehyde at room temperature for 10 min to cross-link DNA. And then washed and resuspended in 400 μl SDS lysis buffer. After 10 min incubation on ice, 600 μl ChIP dilution buffer (0.01% SDS, 1.1% Triton X-100, 1.2 mM EDTA, 16.7 mM Tris-HCl/pH 8.0 and 16.7 mM NaCl, protease inhibitors) was added and genomic DNA was sheared by sonication to 300–1000 bp long. After lysates were preincubated with protein A-agarose conjugated normal rabbit IgG. Anti-Nrf1 antibody was then added into the cell lysates at 4 °C for overnight. IgG was used as the negative control. Specific primer sets were designed to amplify a target sequence within FUNDC1’s promoter. PCR products were analyzed by agarose gel electrophoresis.

### Bioinformatic analysis

The mitophagy PCR array analysis and three-dimensional histogram was performed by the XYbiotech Company (Shanghai, China). The heatmap were made by TBtools software. Volcano Plots were drawed by Graphad prism 7 (San Diego, USA). The Genotype-Tissue Expression (GTEx) Database were analyzed via Gene Expression Profiling Interactive Analysis (GEPIA) webtools. Transcription factor binding profiles were performed through Jaspar website (http://jaspar.genereg.net/).

### Statistical analysis

All the studies were designed to generate groups of equal sample size, using randomization and blinded analysis. Group size is the number of independent values and statistical analysis was undertaken only for studies when these independent values with *n* ≥ 5. To control for unwanted sources of variation, data were normalized. The data are presented as the means ± SEM. For the comparison between two groups, a student’s *t* test was used for normally distributed data, whereas Mann–Whitney *U* test was applied for data with non-normal distribution. Multiple comparisons between the groups were assessed with one-way ANOVA, followed by Turkeys multiple comparison tests. Post hoc tests were run only if *F* achieved *P* < 0.05 and there was no significant variance inhomogeneity. Spearman’s correlation coefficient was used to estimate the association of ALDH2 levels and several factors of interest. All quantification analysis were performed by GraphPad Prism 7.00 (RRID: SCR_002798, San Diego, CA, USA). *P* < 0.05 was considered statistically significant. No data were excluded from any study.

## Results

### α-LA attenuates TAC-induced LV hypertrophy and heart failure in WT mice

**Fig. 1:**
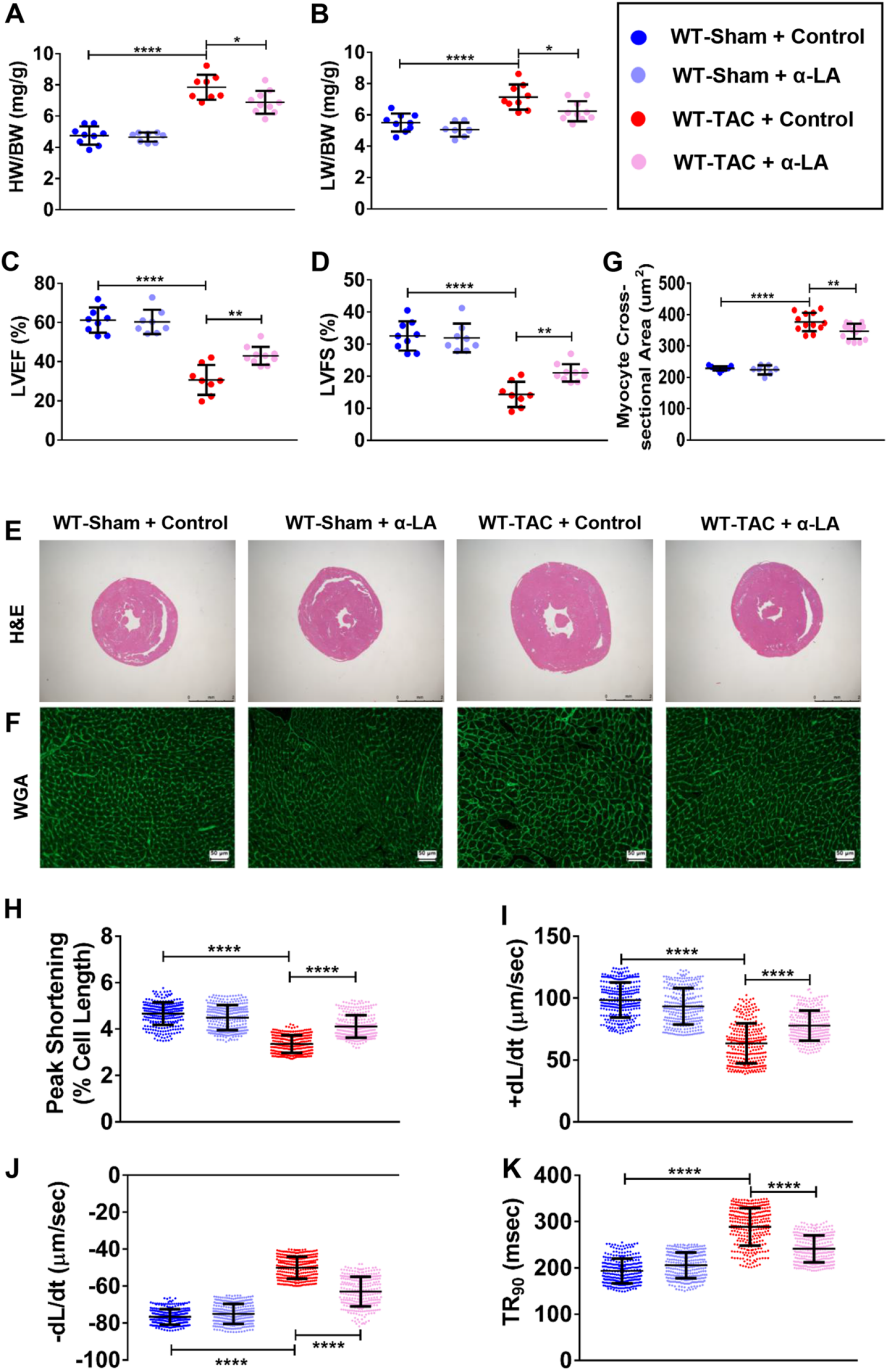
α-LA attenuates TAC-induced LV hypertrophy and heart failure in wild-type mice. **a** Quantitative analysis of heart-to-body weight ratio (HW/BW) (*n* = 8–10 mice per group); **b** Quantitative analysis of lung-to-body weight ratio (LW/BW) (*n* = 8–10 mice per group); **c** left ventricular ejection fraction (LVEF, %) (*n* = 8–10 mice per group); **d** left ventricular fractional shortening (LVFS, %) (*n* = 8–10 mice per group); **e** hematoxylin-eosin (H&E, scale bar = 2 mm) staining; **f** wheat germ agglutinin (WGA, scale bar = 50 μm) staining; **g** quantitative analysis of cardiomyocyte cross-sectional areas (CSA) (*n* = 7–15 mice per group); **h** cardiomyocyte peak shortening (PS, normalized to resting cell length) (*n* = 300 cells per group); **i** maximal velocity of shortening (+d*L*/d*t*) (*n* = 300 cells per group); **j** maximal velocity of relengthening (−d*L*/d*t*) (*n* = 300 cells per group); **k** time-to-90% relengthening (TR90) in isolated cardiomyocytes (*n* = 300 cells per group). Mean ± SEM, *P < 0.05, **P < 0.01, ****P < 0.0001. Statistical analysis was carried out by a one-way ANOVA analysis followed by Tukey’s test for post hoc analysis.

In order to study the cardio-protective effect of α-LA, we treated TAC-operated WT mice with α-LA (0.2% wt/wt) or vehicle (normal saline) for 4 weeks. Significantly increased heart weight, lung weight (Fig. 1a, b), and reduced LV ejection fraction and fractional shortening were evidenced in WT TAC mice (Fig. 1c, d). α-LA significantly attenuated TAC-induced LV hypertrophy and lung weight (Fig. 1a, b) and improved cardiac function (Fig. 1c, d) in WT TAC mice. Moreover, LVESD, LV end-diastolic diameter (LVEDD), and LV diastole posterior wall thickness (LVPWD) were increased in WT mice post TAC surgery, while α-LA decreased TAC-induced increase of LVESD, LVEDD, and LVPWD (Supplementary Fig. S1A). Based on histological analysis, α-LA decreased TAC-induced increase of LV hypertrophy (Fig. 1e–g). Consistently, the quantitative real-time PCR (qRT-PCR) results showed that α-LA reduced these hypertrophic pathological phenotypes were accompanied by the downregulation of hypertrophic genes, including atrial natriuretic polypeptide (ANP), brain natriuretic peptide (BNP), myosin heavy chain beta (β-Mhc), and fibrotic genes including connective tissue growth factor (Ctgf), collagen1a1 (Col1a1), and collagen3a1 (Col3a1) (Supplementary Fig. S1B). Similarly, cardiomyocytes contractile parameters were significantly improved by α-LA treatment in pressure overload mice (Fig. 1h–k).

### α-LA reduces TAC-induced cardiomyocyte apoptosis and cardiac fibrosis in WT mice

**Fig. 2:**
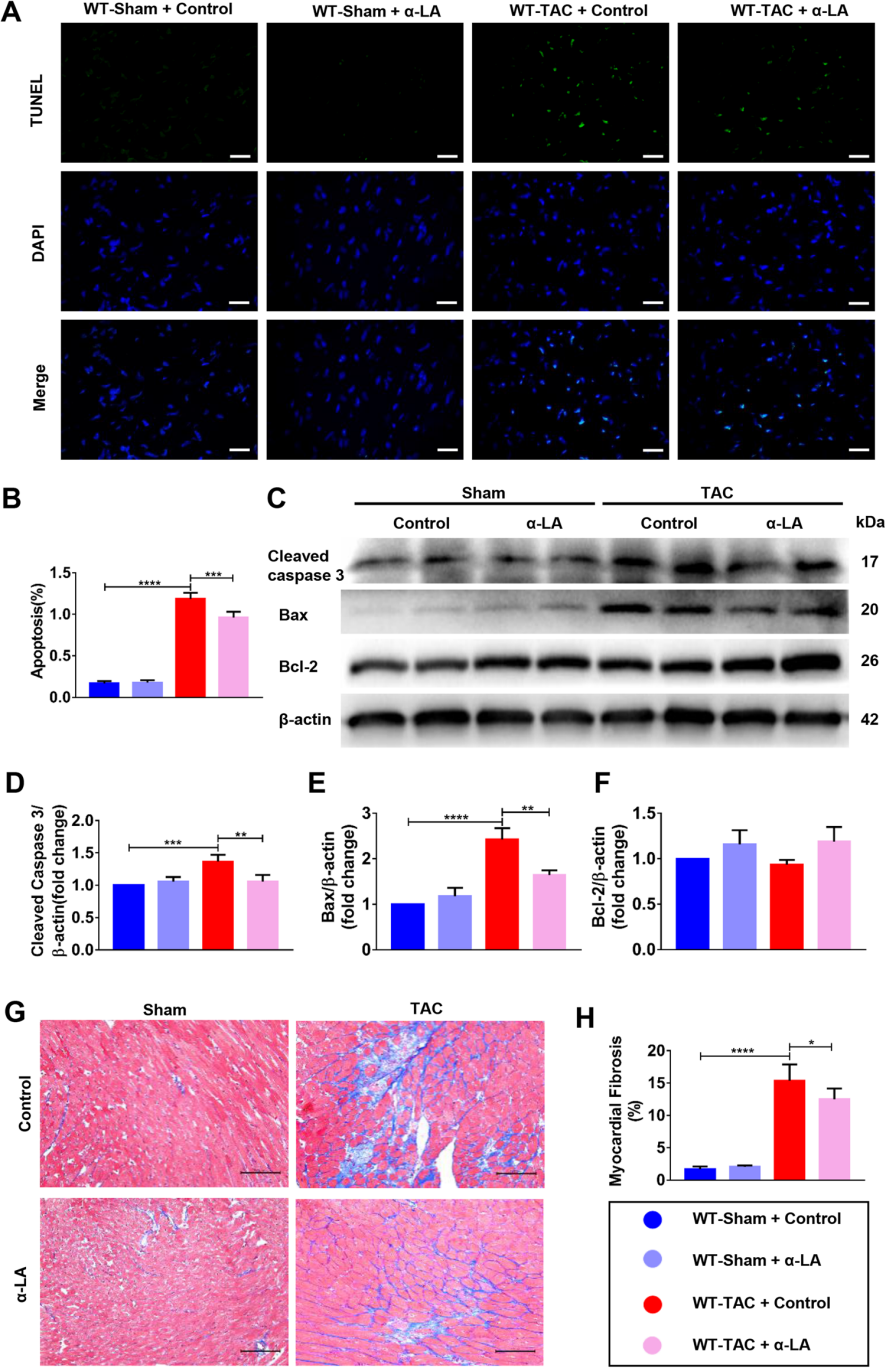
α-LA reduces TAC-induced cardiomyocyte apoptosis and cardiac fibrosis in WT mice. **a** Terminal dexynucleotidyl transferase (TdT)-mediated dUTP nick end labeling staining(TUNEL, 400×, scale bar = 20 μm); **b** quantitative analysis of myocardial apoptosis (*n* = 6 mice per group); **c** Western blots of cleaved caspase 3, Bax, Bcl-2, and β-actin (loading control); **d**–**f** Quantitative analysis of expressions of cleaved caspase 3/β-actin, Bax/β-actin, Bcl-2/β-actin (*n* = 6 mice per group); **g** Masson’s Trichome staining (scale bar = 100 μm); **h** quantitative analysis of myocardial fibrosis (*n* = 6 mice per group). Mean ± SEM, *P < 0.05, **P < 0.01, ***P < 0.001, ****P < 0.0001. Statistical analysis was carried out by a one-way ANOVA analysis followed by Tukey’s test for post hoc analysis.

To investigate potential causes of cardiac dysfunction after TAC, we analyzed cardiomyocyte apoptosis. LV sections were stained for TUNEL to determine the rate of cardiomyocyte apoptosis. A noticeable increase in cardiomyocyte apoptosis was observed after TAC in WT mice (Fig. 2a, b) and α-LA administration substantially reduced TAC-induced apoptosis (Fig. 2a, b). Next, we investigated the molecular mediators of apoptosis in WT mice with TAC. TAC markedly increased cleaved caspase-3 and Bax, while α-LA treatment significantly reduced cleaved caspase-3 and Bax expression post TAC in WT mice, but Bcl-2, an anti-apoptotic protein was not affected by α-LA treatment in the TAC model (Fig. 2c–f). The results indicated that α-LA might inhibit the key proapoptotic mediators and then decreased myocardial apoptosis. As cardiac fibrosis is another key process in the progression of pathological cardiac remodeling, we assessed the level of fibrosis in mice after TAC with or without additional α-LA treatment. In agreement with above results, a significant increase in cardiac fibrosis was observed 4 weeks after TAC and α-LA treatment markedly reduced cardiac fibrosis (Fig. 2g, h). Taken together, α-LA demonstrated both anti-apoptosis and anti-fibrotic properties in TAC models in WT mice.

### α-LA reduces TAC-induced mitochondrial dysfunction and increases ALDH2 activity and expression

**Fig. 3:**
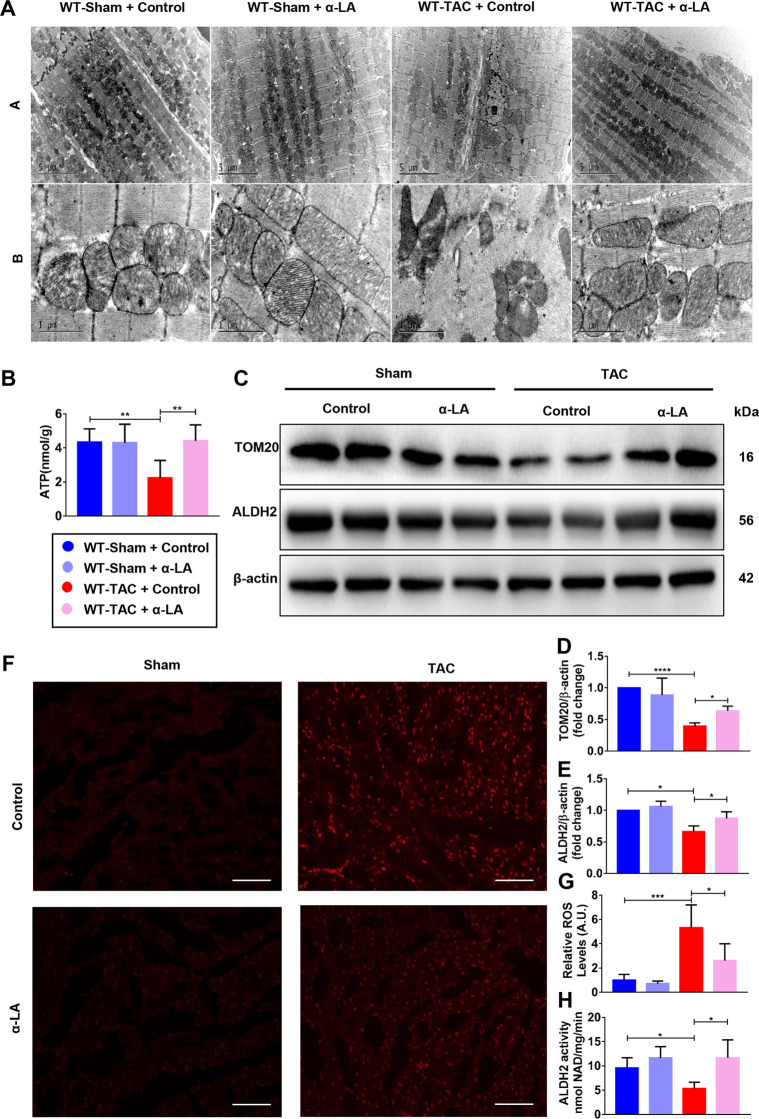
α-LA reduces TAC-induced mitochondrial dysfunction and increases ALDH2 activity and expression. **a** Transmission electron microscopy (TEM) images of left ventricular, Up: scale bar = 5 μm, down: scale bar = 1 μm; **b** quantitative analysis of ATP production of left ventricular; **c**–**e** Western blots of TOM20, ALDH2, and β-actin (loading control) and quantitative analysis of expressions of TOM20/β-actin, ALDH2/β-actin (*n* = 6 mice per group); **f** dihydroethidium (DHE, 200×, scale bar = 50 μm) staining; **g** quantitative analysis of myocardial ROS levels (*n* = 6 mice per group); **h** quantitative analysis of myocardial ALDH2 activity (*n* = 6 mice per group). Mean ± SEM, *P < 0.05, **P < 0.01, ***P < 0.001. Statistical analysis was carried out by a one-way ANOVA analysis followed by Tukey’s test for post hoc analysis.

Our previous study indicated pressure overload-induced heart injury is often accompanied by mitochondrial damage^5^, the changes of mitochondrial morphology was observed with TEM. In the absence of α-LA treatment, significant difference was found in myocardial ultrastructure between sham and TAC group, and the mitochondria became wizened and cristae became disorganized in mitochondria of myocardial tissue from the TAC mice. However, α-LA treatment restored mitochondrial amount and morphology to some extent in TAC mice (Fig. 3a). To confirm the improvement in mitochondrial function, we measured mtDNA and found mtDNA copy number decreased on 4 weeks after TAC but restored after the administration of α-LA (Supplementary Fig. S1C). In addition, TAC surgery significantly decreased total ATP production when compared to the sham group, primarily due to a significant damage in myocardial mitochondria. α-LA also showed a trend to increase total ATP production due to a significant improvement in mitochondrial quality in TAC hearts (Fig. 3b). Western blot analysis revealed a decrease in mitochondrial marker TOM20 and ALDH2 expression in hearts of TAC mice as compared with sham group (Fig. 3c–e). It is known that the primary function of ALDH2 is detoxifying reactive aldehydes and inadequate acetaldehyde could increase the generation of oxidative stress^8^. So we wanted to see if oxidative stress was also involved in this pathological process. We detected ROS level by dihydroethidium (DHE) staining. As shown in Fig. 3f, g, ROS accumulation was significantly increased after TAC surgery and decreased in α-LA treatment group. SOD2, as a tetramer, is initially synthesized containing a leader peptide, which targets this manganese-containing enzyme exclusively to the mitochondrial spaces. As show in Supplementary Fig. S1D, we can see SOD2 was downregulated in TAC group, and α-LA could reverse SOD2 expression. Previous study reported that ALDH2 active site has redox-sensitive sulfhydryl group, so when the oxygen free radicals in the body increased, the disulfide bonds at the active site would be formed and became inactivated^10^. As expected, by measuring the conversion of propionaldehyde, ALDH2 activity was decreased by 44% in TAC group compared with the sham group and α-LA treatment significantly improved mitochondrial ALDH2 activity (Fig. 3h). These observations indicated that α-LA restored ALDH2 activity as well as increased ALDH2 expression and protected the heart from pressure overload-induced mitochondrial injury in WT mice.

### FUNDC1 modulates α-LA effects in TAC-induced heart failure model

**Fig. 4:**
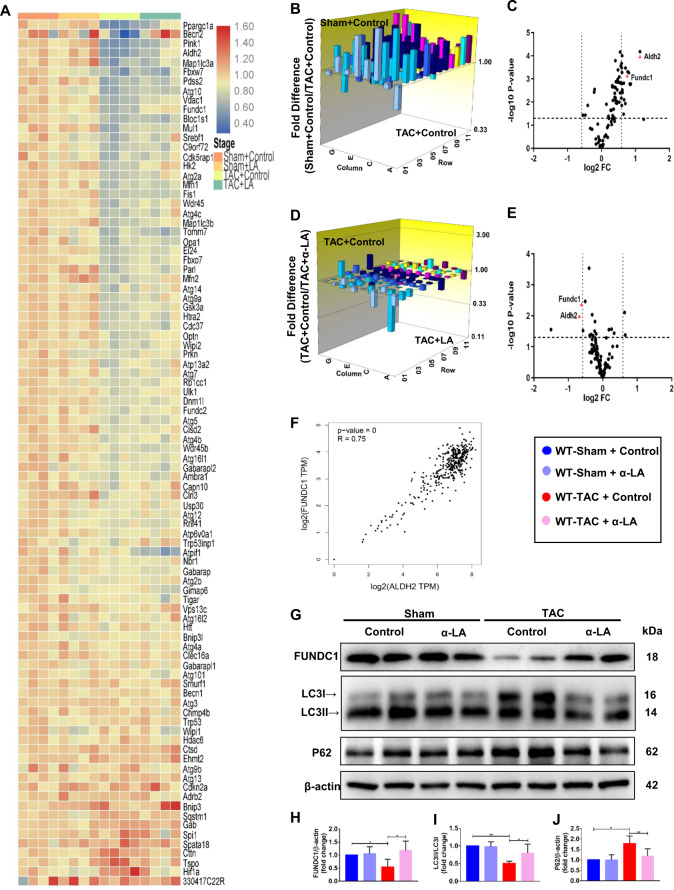
FUNDC1 modulates α-LA effects in TAC-induced heart failure model. **a** Heatmap of different groups in mRNA array; **b** three-dimensional diagram of mitophagy related genes when compared Sham+Control group with TAC + Control group; **c** volcano plot graphs of mPCR array. Graph shows the log2 of fold change of ALDH2 and FUNDC1 gene expression between Sham+Control group and TAC + Control group versus *P* value from the *t* test; **d** three-dimensional diagram of mitophagy related genes expression when compared TAC + Control group with TAC + LA group; **e** volcano plot graphs of mPCR array. Graph shows the log2 of fold change of ALDH2 and FUNDC1 gene expression between TAC + Control group and TAC + LA group versus *P* value from the *t* test; **f** correlation between FUNDC1 and ALDH2 expression in GTEx database; **g** Western blots of FUNDC1, LC3, P62, and β-actin (loading control); **h**–**j** quantitative analysis of expressions of FUNDC1/β-actin, LC3II/LC3I, P62/β-actin (*n* = 6 mice per group). Mean ± SEM, *P < 0.05, **P < 0.01. Statistical analysis was carried out by a two-tailed Student’s *t* test or one-way ANOVA analysis followed by Tukey’s test for post hoc analysis.

To confirm whether mitophagy is involved in the pathological process of cardiac hypertrophy, left ventricles from sham or TAC mice were also analyzed for 96 associated genes expression by mitophagy related microarray (Fig. 4a; Supplementary Table S1) in which FUNDC1 was one of the significantly changed genes. As shown in Fig. 4b, c and Supplementary Fig S1E, 4 weeks of pressure overload caused significant difference in ALDH2 and FUNDC1 gene expression when compared to the sham group. Remarkably, α-LA treated TAC mice displayed a significant increase (≈1.6-fold) in ALDH2 and (≈1.5-fold) in FUNDC1 gene expression compared with vehicle treated TAC mice (Fig. 4d, e). In addition, there was a significant positive correlation between FUNDC1 and ALDH2 in normal left ventricle and atrial appendage tissue through the GTEx Database (Fig. 4f). Western blot assay showed that FUNDC1 protein expression was remarkably increased post α-LA treatment in TAC mice (Fig. 4g, h). We also did FUNDC1 immunohistochemistry staining and found the expression was downregulated in TAC group but reversed by α-LA, which was consistent with our microarray and western blot data (Supplementary Fig. S1F). As previously supported, our data also depicted that pressure overload significantly suppressed myocardial autophagy (LC3II-to-LC3I ratio) and affected autophagy flux as evidenced by an increase of invariant autophagy adapter protein p62, while α-LA treatment prevented this pathological process (Fig. 4g, i, j). Overall, our data suggested that the anti-hypertrophic activity of α-LA was associated with changes in the expression of FUNDC1 related mitophagy in pressure overload hearts.

### α-LA treatment improves cardiac function in TAC mice via ALDH2-dependent activation of FUNDC1

**Fig. 5:**
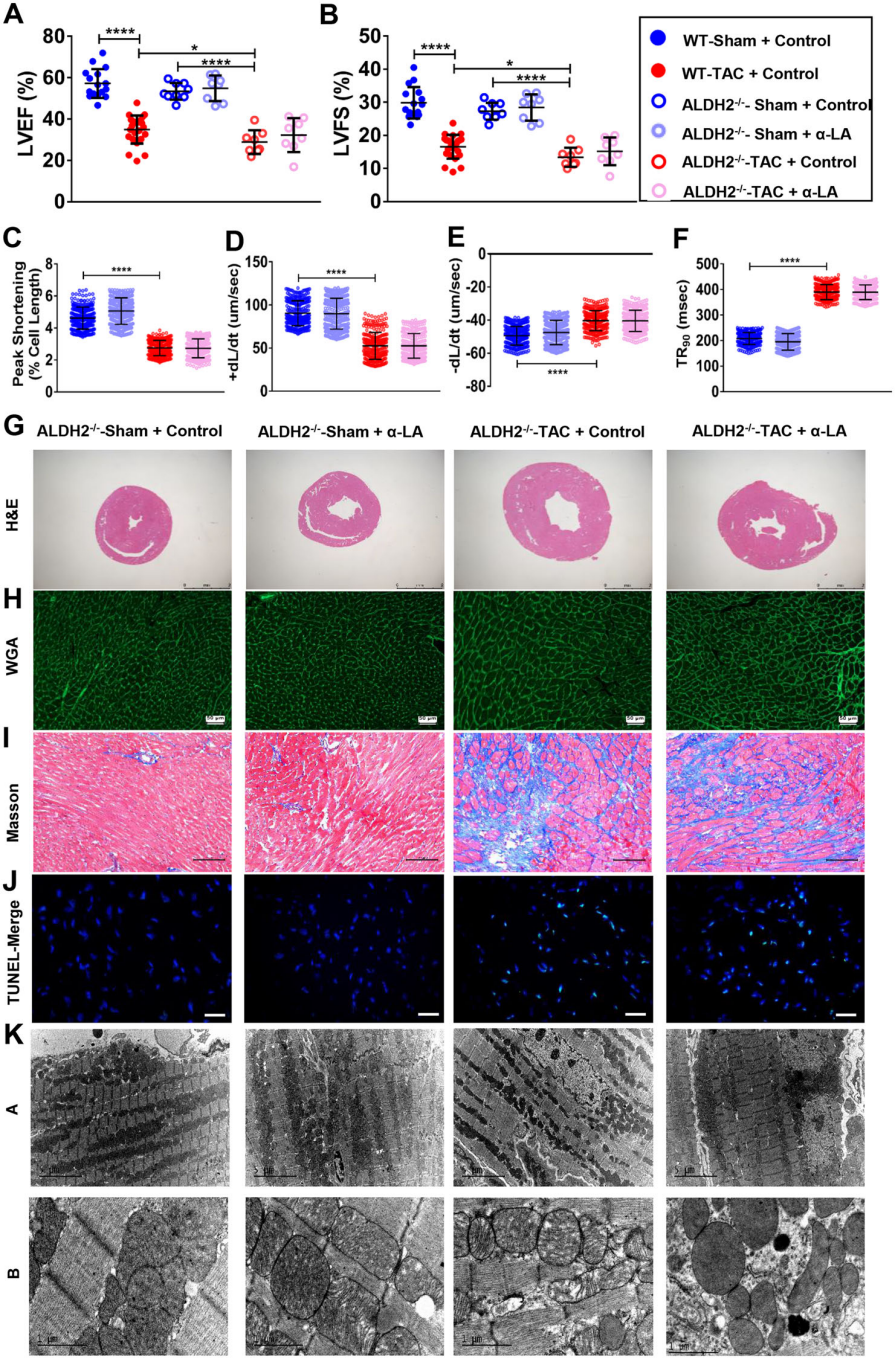
α-LA treatment improves cardiac function in TAC mice via ALDH2-dependent activation of FUNDC1. **a** Left ventricular ejection fraction (LVEF, %) (*n* = 8–20 mice per group); **b** left ventricular fractional shortening (LVFS, %) (*n* = 8–20 mice per group); **c** cardiomyocyte peak shortening (PS, normalized to resting cell length) (*n* = 300 cells per group); **d** maximal velocity of shortening (+d*L*/d*t*) (*n* = 300 cells per group); **e** maximal velocity of relengthening (−d*L*/d*t*) (*n* = 300 cells per group); **f** time-to-90% relengthening (TR90) in isolated cardiomyocytes (*n* = 300 cells per group); **g** hematoxylin–eosin (H&E, scale bar = 2 mm) staining; **h** wheat germ agglutinin (WGA, scale bar = 50 μm) staining; **i** Masson’s Trichome staining (scale bar = 100 μm); **j** terminal dexynucleotidyl transferase (TdT)-mediated dUTP nick end labeling staining (TUNEL, 400×, scale bar = 20 μm); **k** transmission electron microscopy (TEM) images of left ventricular, Up: scale bar = 5 μm, Down: scale bar = 1 μm. Mean ± SEM, *P < 0.05, ****P < 0.0001. Statistical analysis was carried out by a one-way ANOVA analysis followed by Tukey’s test for post hoc analysis.

Next, to verify if the observed effects of α-LA in TAC mice is led to ALDH2 dependent or not, the effects of α-LA were examined in ALDH2 knockout mice (Supplementary Fig. S2A). In normal condition, there is no difference in echocardiography between WT mice and ALDH2^−/−^ mice (Fig. 5a, b, Supplementary Fig. S2B), but ALDH2 deficiency exacerbated the decrease of LVEF and LVFS as well as enlargement of dimensions measurements after TAC surgery when compared with WT TAC mice (Fig. 5a, b; Supplementary Fig. S2B). But, α-LA did not improve cardiac function after TAC surgery in ALDH2^−/−^ mice (Fig. 5a, b, Supplementary Fig. S2B). Cardiomyocyte contractile results were also not improved by α-LA (Fig. 5c–f). Deficiency of ALDH2 resulted in worse cardiac hypertrophy and fibrosis in pressure overload mice (Fig. 5g–i, Supplementary Fig. S2C–E) both in α-LA treated group and vehicle treated group, suggesting that the attenuating-remodeling effects of α-LA are largely mediated by ALDH2. As shown in Fig. 5j and Supplementary Fig. S2F–H, TAC surgery induced severe myocardial apoptosis in ALDH2^−/−^ mice myocardium. Again, α-LA treatment didn’t supply a significant anti-apoptosis role in ALDH2^−/−^ TAC mice. At the same time, α-LA did not affect ROS accumulation in ALDH2^−/−^ TAC mice as compared with vehicle treated ALDH2^−/−^ TAC mice (Supplementary Fig. S2I, J). In mitochondrial aspect, there was also no difference in ALDH2^−/−^ TAC mice treated with or without α-LA, similar myocardial mitochondria swelling changes and disorganized cristae were observed between the two groups (Fig. 5k). Since FUNDC1 signaling was shown to be involved in α-LA induced beneficial effects in WT TAC mice, we next investigated whether the interaction of α-LA and FUNDC1 signaling was dependent of ALDH2. We found that the mitophagy related protein FUNDC1, LC3B, P62 remained unchanged in ALDH2^−/−^ TAC mice post α-LA treatment as compared with vehicle treated ALDH2^−/−^ TAC mice (Supplementary Fig. S2K–N). Taken together, these data suggested that α-LA prevented TAC-induced FUNDC1 inactivation in an ALDH2-dependent manner.

### α-LA attenuates angiotensin II-induced hypertrophic response in NRCMs in an ALDH2-dependent manner

**Fig. 6:**
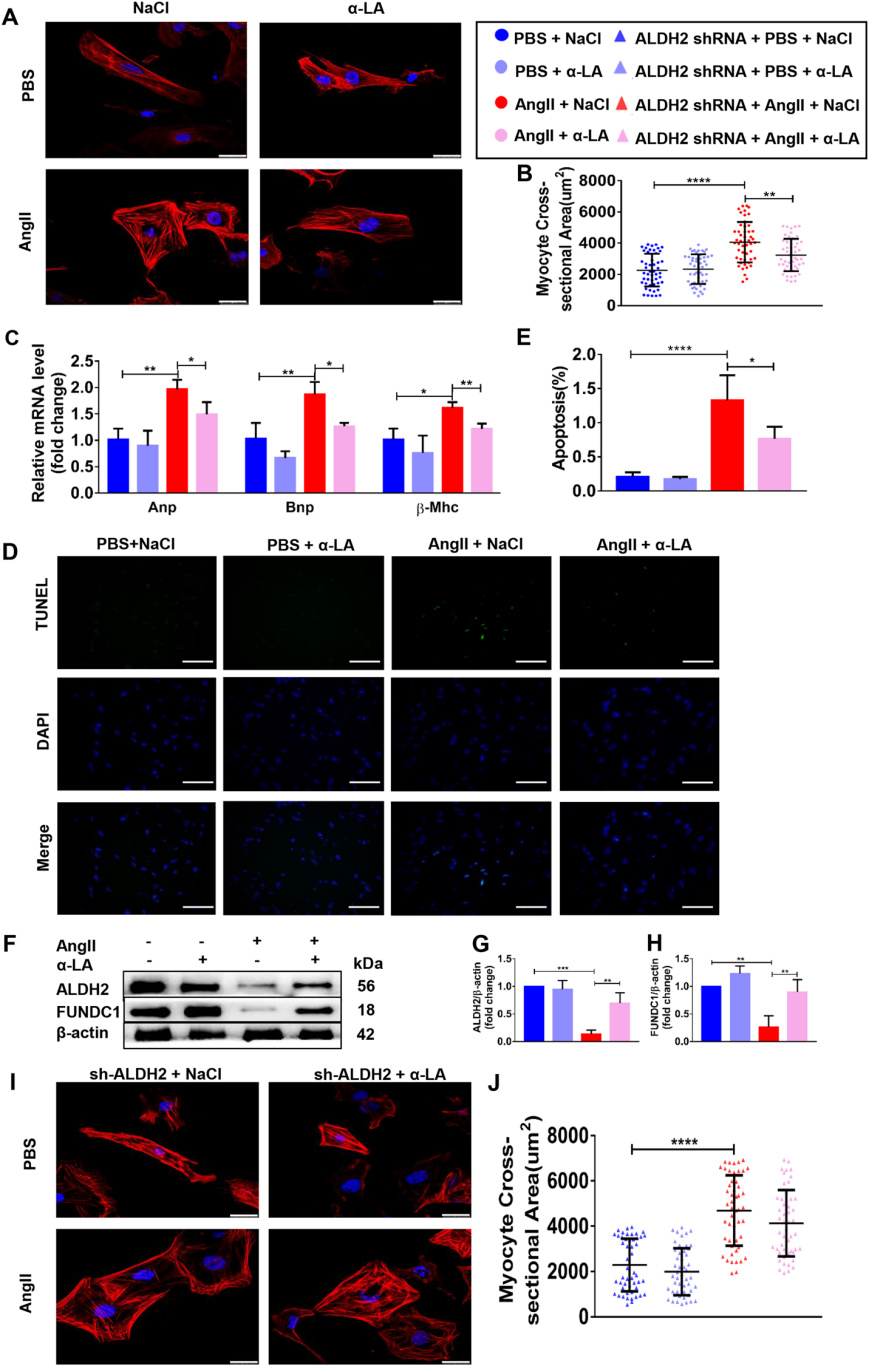
α-LA attenuates angiotensin II-induced hypertrophic response in neonatal rat cardiomyocytes (NRCMs) in an ALDH2-dependent manner. **a** TRITC Phalloidin staining (scale bar = 25 μm); **b** quantitative analysis of NRCM cell surface area using measurement from 50 cells per group; **c** quantitative real-time PCR (qRT-PCR) analyzing the mRNA levels of hypertrophic (Anp, Bnp, and β-Mhc) genes (*n* = 6 samples per group); **d** terminal dexynucleotidyl transferase (TdT)-mediated dUTP nick end labeling staining (TUNEL, 200×, scale bar = 50 μm); **e** quantitative analysis of myocardial apoptosis (*n* = 6 samples per group); **f** Western blots of ALDH2, FUNDC1 and β-actin (loading control); **g**, **h** Quantitative analysis of expressions of ALDH2/β-actin, FUNDC1/β-actin (*n* = 6 samples per group); **i** TRITC Phalloidin staining (scale bar = 25 μm); **j** quantitative analysis of NRCM cell surface area using measurement from 50 cells per group. Mean ± SEM, *P < 0.05, **P < 0.01, ***P < 0.001, ****P < 0.0001. Statistical analysis was carried out by a one-way ANOVA analysis followed by Tukey’s test for post hoc analysis.

In order to find out the molecular mechanisms of α-LA-mediated protection of cardiac hypertrophy, we firstly established a cell culture model in neonatal rat ventricular myocytes (NRCMs). NRCMs were treated with Angiotensin II (AngII, 1 µM) to induce hypertrophy. AngII treatment for 48 h triggered a hypertrophic response, as shown by significantly increased NRCMs surface area (Fig. 6a, b). In addition, AngII treatment significantly increased ANP (*p* < 0.01), BNP (*p* < 0.01), and β-MHC (*p* < 0.05) gene expression (Fig. 6c). AngII treatment also increased NRCMs apoptosis (*p* < 0.0001) (Fig. 6d, e). Further, to check the role of α-LA on AngII induced hypertrophy, NRCMs were pre-treated with α-LA (10 μM) or PBS for 1 h followed by AngII for 48 h. α-LA markedly reduced AngII-induced increase on NRCMs surface area, hypertrophic genes expression as well as cell apoptotic response (Fig. 6a–e). Moreover, to study the intermediate signaling components, western blot analysis of the total cell lysates from NRCMs was performed after AngII stimulation in the presence or absence of α-LA pretreatment for protein expression of ALDH2 and FUNDC1. AngII significantly downregulated ALDH2 and FUNDC1 protein expression, which could be markedly upregulated after α-LA treatment (Fig. 6f–h). Next, we knocked down ALDH2 in NRCMs cells by lentivirus (Supplementary Fig. 3A), the AngII-induced hypertrophic response could not be reversed by α-LA in ALDH2-deficient NRCMs cells, suggesting that the anti-hypertrophic responses of α-LA was mediated by ALDH2 (Fig. 6i, j).

### ALDH2 protects angiotensin II-induced hypertrophic response via Nrf1-FUNDC1-dependent pathway

**Fig. 7:**
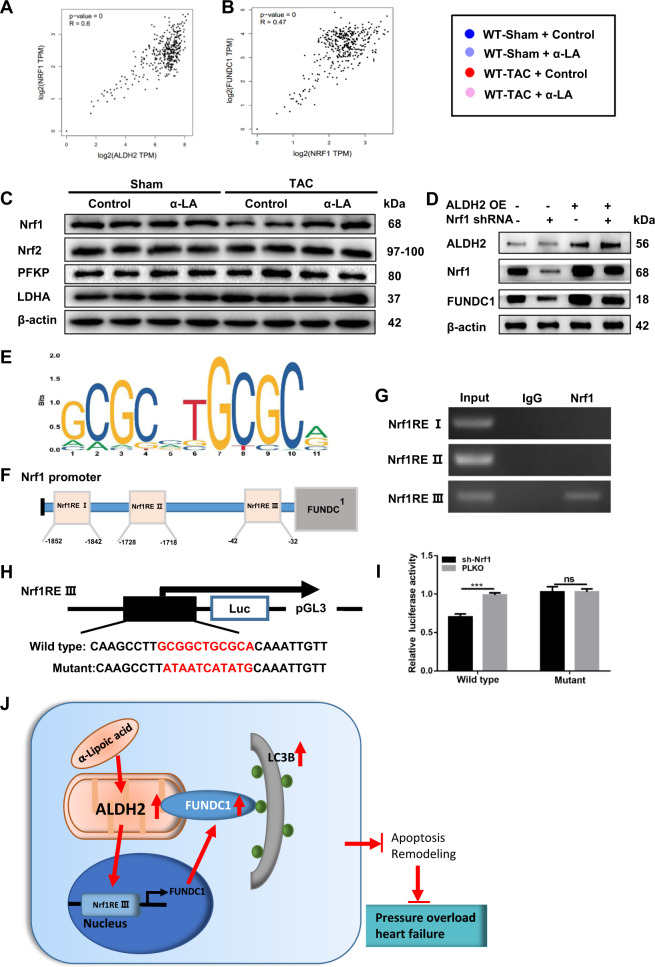
ALDH2 protects angiotensin II-induced hypertrophic response via Nrf1-FUNDC1-dependent pathway. **a**, **b** Correlation between ALDH2 and Nrf1 expression as well as FUNDC1 and Nrf1 in GTEx database; **c** Western blots of Nrf1, Nrf2, PFKP, LDHA, and β-actin in tissue (loading control) (*n* = 6 samples per group); **d** Western blots of ALDH2, FUNDC1, Nrf1, and β-actin (loading control) in NRCMs after transfection (*n* = 6 samples per group); **e** depiction of the putative Nrf1-binding site located at FUNDC1 promoter; **f** predicted potential Nrf1REs; **g** chromatin immunoprecipitation (ChIP) assay in NRCMs; **h** construction pGL3 luciferase reporter vector with FUNDC1 promoter; **i** luciferase assay in NRCMs (*n* = 6 samples per group); **j** schematic diagram depicting the role of α-LA in pressure overload-induced changes in ALDH2 and mitophagy. Pressure overload suppresses expression and activity of myocardium ALDH2, which participates in pressure overload-induced inhibition of mitophagy receptor FUNDC1 and LC3II. Besides, the beneficial effects of α-LA in pressure overload heart failure was mediated by a novel ALDH2–Nrf1–FUNDC1 signaling pathway in which procedure Nrf1 modulates the FUNDC1 expression via directly binding to the 5′ promoter of FUNDC1. Mean ± SEM, ***P < 0.001. Statistical analysis was carried out by a two-tailed Student’s *t* test analysis.

Previous reports have documented the involvement of Nrf1 transcription regulation signaling in both mitochondrial metabolism and mitophagy processes^28^. Moreover, there is a significant positive correlation between ALDH2 and Nrf1, as well as Nrf1 and FUNDC1 in normal left ventricle and atrial appendage tissue through the GTEx Database (Fig. 7a, b). Therefore, we investigated whether Nrf1 had interaction on ALDH2 and FUNDC1 signaling. Western blot results showed that Nrf1 was significantly decreased after TAC surgery in WT mice, while α-LA treatment increased the expression of Nrf1 to normal level (Fig. 7c, Supplementary Fig. S3B–E). To examine if ALDH2 regulated FUNDC1 via Nrf1, we assessed the expression of FUNDC1 by overexpressing ALDH2 and knocking down Nrf1 (Supplementary Fig. 3F, G). After ALDH2 overexpression, both Nrf1 and FUNDC1 increased. But if we knocked down Nrf1, ALDH2 overexpression failed to upregulate FUNDC1 signaling, suggesting that ALDH2-regulated FUNDC1 via Nrf1 (Fig. 7d, Supplementary Fig. 3H). Mechanistically, as a member of the transcription factors, Nrf1 may function via binding to the Nrf1-response-elements (Nrf1REs) located on its target genes (here is FUNDC1) promoter regions to modulate their expression. To test this hypothesis, we firstly searched for potential Nrf1REs (Fig. 7e) using Jaspar webtools (http://jaspar.genereg.net/) and found three putative Nrf1REs within the proximal 2 kb promoter region (Fig. 7f, Supplementary Table S4). ChIP assay results showed that Nrf1RE III, but not Nrf1RE I or II, served as the potential Nrf1 binding sites (Fig. 7g). Then, we cloned this 2 kb FUNDC1 promoter into pGL3-basic luciferase reporter vector (Fig. 7h). The luciferase assay results revealed that Nrf1 knockdown significantly decreased the luciferase reporter activity in WT group, while mutation group failed to change the luciferase reporter activity (Fig. 7i), suggesting Nrf1 modulated the FUNDC1 expression via binding to its promoter. Taken together, results from Fig. 7d–i suggested that Nrf1 could modulate the FUNDC1 expression via directly binding to the 5′ promoter of FUNDC1.

## Discussion

In the present study, we showed that alpha-lipoic acid therapy attenuated pressure overload-induced cardiac hypertrophy and remodeling and improved the cardiac function in TAC mice via ALDH2-dependent manner. Mechanistically, alpha-lipoic acid activates FUNDC1 signaling via Nrf1 pathway. To our best knowledge, this is the first study evaluating the molecular mechanisms of alpha-lipoic acid in the TAC induced heart failure model (Fig. 7j).

As an efficient metabolic enzyme in mitochondria, growing evidence supports an important role of ALDH2 in various forms of heart failure^29^. Studies showed that pharmacological activation or overexpression of ALDH2 provided cardio-protection against ischemia and atherosclerosis by detoxification of reactive aldehydes^30,31^, however, another study showed that overexpression of aldehyde dehydrogenase 2 could even exacerbate cardiac remodeling in the setting of pressure overload^32^. The impact and related mechanisms of α-LA, a known ALDH2 activator, in pressure overload induced cardiac remodeling and heart failure are not fully understood. Our results revealed that 4 weeks treatment with α-LA restored the expression and activity of ALDH2 in WT TAC mice and attenuated cardiac remodeling and improved cardiac function in this model. We also found α-LA could reduce TAC-induced cardiac fibrosis in WT mice. Based on previous studies, a-LA could reduce pathologically-induced fibrosis, such as radiation-induced injury^33,34^ and carbon tetrachloride-induced liver fibrosis^35^, but not a direct antifibrotic effect, which were consistent with our results. ROS are well-known for their role in mediating both physiological and pathophysiological signal transduction. In fact, ROS is highly reactive in cardiomyocyte hypertrophy and even in the activation of cardiac fibroblasts^36^. Clinical and experimental studies have provided enough evidences that excess ROS can cause myocardial remodeling, including contractile dysfunction and structural alterations^37^. Of cause, ischemia and hypoxia happens in TAC group which could further increase ROS triggered programmed cell death by the mechanism of apoptosis or autophagy. Maybe this is one reason why the phenotype only occurs in TAC group. The increase of ALDH2 activity can be explained as follows: ALDH2 has a redox-sensitive sulfhydryl group at the active site, in some pathological processes, increased reactive oxygen species could promote the formation of disulfide bonds at this active site, as a consequence, the ALDH2 is inactivated. α-LA can break the binding between disulfide bond and the redox-sensitive sulfhydryl group at the active site, thus prevent the ALDH2 inactivation.

Mitophagy is a special autophagy process capable of clearing damaged mitochondria. Although increasing evidence suggests that dysregulation of mitophagy can induce heart failure^38–40^, the underlying regulatory mechanisms of mitophagy in heart failure have not been fully elucidated. FUNDC1 (FUN14 domain containing 1) is a mitophagy receptor of LC3II, which localizes in the outer mitochondrial membrane^41^. The combination of LC3II and FUNDC1 promotes mitochondrial lysosomes and increases mitophagy. Moreover, it has been reported that FUNDC1-related mitophagy could also attenuate cardiomyocyte oxidative stress, sustain mitochondrial membrane potential and prevent mitochondrial apoptosis in myocardial ischemia reperfusion injury^41,42^. Nrf1, which belongs to the Cap-N-Collar family of regulatory proteins, is a transcription factor responsible for the activation of expression of many factors involved in energy metabolism, cellular respiration, and oxidative stress response^28^. ALDH2 activation was reported to suppress phosphatase and tensin homolog-induced putative kinase 1 (PINK1)/Parkin expression, therefore regulating mitophagy process in myocardial ischemia/reperfusion (I/R)^43^. However, it is not clear whether ALDH2 can regulate FUNDC1 or whether ALDH2 can regulate mitophagy under the pathological conditions of pressure overload. In our study, we found that FUNDC1 was downregulated in pressure-overload induced heart failure. α-LA treatment could activate FUNDC1 signaling and enhance mitophagy in TAC mice in an ALDH2 dependent manner. Moreover, ChIP assay and luciferase assay results showed that ALDH2 activated FUNDC1 via activating Nrf1, in that Nrf1 could directly bind to the 5′ promoter of FUNDC1. From our microarray data, we can see some mitochondria and autophagy related genes, such as Atpif1, BECN2 and BNIP3 changed dramatically, which means α-LA and ALDH2 may also function through these genes. Previous study found Atpif1 and the ATP synthase could be novel components of the Pink1–Parkin mitophagy pathway in cultured cells^44^. BECN2, a novel Atg6/Beclin family member, functions in autophagy and metabolism^45^. Chen et al.^46^ also found α-LA could protect against acute nephrotoxicity through decreasing BNIP3. Those above studies implied that α-LA and mitophagy is a complex process and we will investigate those genes in our further research.

Epidemiological studies demonstrated that more than 500 million people worldwide, mostly in East Asia, have a G-to-A point mutation in their ALDH2 gene^47^. This mutation results in a glutamic acid-to-lysine substitution at residue 487 (E487K) of the human ALDH2 protein, ALDH2 activity in this population is reduced more than 50% as compared to people with the WT ALDH2. Since the beneficial effects of α-LA in the pressure overload induced cardiac failure is ALDH2 dependent, the efficacy of α-LA might be limited in population with a G-to-A point mutation of the ALDH2 gene. Thus, individualized treatment option on background of genotyping is essential on the decision making regarding the use of α-LA in treating pressure-overload induced heart failure in the daily clinical practice.

In summary, our results suggest that α-LA could attenuate pressure overload induced cardiac injury in an ALDH2 dependent manner, clinical studies are warranted to verify if α-LA could be a potential useful therapeutic option to treat heart failure patients with pressure overload and with WT ALDH2.

## Supplementary information

Supplementary Information

Supplementary Figure 1

Supplementary Figure 2

Supplementary Figure 3

Supplementary data1

Supplementary data2

Supplementary data3

Supplementary data4

## Data Availability

The datasets used during the study are available from the corresponding author on reasonable request.

## References

[1] Bui, A. L., Horwich, T. B. & Fonarow, G. C. Epidemiology and risk profile of heart failure. *Nat. Rev. Cardiol.* **8**, 30–41 (2011). 21060326 10.1038/nrcardio.2010.165 PMC3033496

[2] Bayeva, M., Gheorghiade, M. & Ardehali, H. Mitochondria as a therapeutic target in heart failure. *J. Am. Coll. Cardiol.* **61**, 599–610 (2013). 23219298 10.1016/j.jacc.2012.08.1021 PMC3594689

[3] Marunouchi, T. & Tanonaka, K. Cell death in the cardiac myocyte. *Biol. Pharm. Bull.* **38**, 1094–1097 (2015). 26235571 10.1248/bpb.b15-00288

[4] Jackson, B. et al. Update on the aldehyde dehydrogenase gene (ALDH) superfamily. *Hum. Genomics* **5**, 283–303 (2011). 21712190 10.1186/1479-7364-5-4-283 PMC3392178

[5] Shen, C. et al. Acetaldehyde dehydrogenase 2 (ALDH2) deficiency exacerbates pressure overload-induced cardiac dysfunction by inhibiting Beclin-1 dependent autophagy pathway. *Biochim. Biophys. Acta* **1852**, 310–318 (2015). 25086229 10.1016/j.bbadis.2014.07.014

[6] Shen, C. et al. Aldehyde dehydrogenase 2 deficiency negates chronic low-to-moderate alcohol consumption-induced cardioprotecion possibly via ROS-dependent apoptosis and RIP1/RIP3/MLKL-mediated necroptosis. *Biochim. Biophys. Acta Mol. Basis Dis.* **1863**, 1912–1918 (2017). 27840306 10.1016/j.bbadis.2016.11.016

[7] Sun, A. et al. Mitochondrial aldehyde dehydrogenase 2 plays protective roles in heart failure after myocardial infarction via suppression of the cytosolic JNK/p53 pathway in mice. *J. Am. Heart Assoc.* **3**, e000779 (2014). 25237043 10.1161/JAHA.113.000779 PMC4323818

[8] Wang, S., Pacher, P., De Lisle, R. C., Huang, H. & Ding, W. X. A mechanistic review of cell death in alcohol-induced liver injury. *Alcohol Clin. Exp. Res.* **40**, 1215–1223 (2016). 27130888 10.1111/acer.13078 PMC5455778

[9] Belmont-Diaz, J. A., Yoval-Sanchez, B., Calleja-Castaneda, L. F., Pardo Vazquez, J. P. & Rodriguez-Zavala, J. S. Alda-1 modulates the kinetic properties of mitochondrial aldehyde dehydrogenase (ALDH2). *FEBS J.* **283**, 3637–3650 (2016). 27521998 10.1111/febs.13833

[10] Wenzel, P. et al. Role of reduced lipoic acid in the redox regulation of mitochondrial aldehyde dehydrogenase (ALDH-2) activity. Implications for mitochondrial oxidative stress and nitrate tolerance. *J. Biol. Chem.* **282**, 792–799 (2007). 17102135 10.1074/jbc.M606477200

[11] He, L. et al. Alpha lipoic acid protects heart against myocardial ischemia-reperfusion injury through a mechanism involving aldehyde dehydrogenase 2 activation. *Eur. J. Pharm.* **678**, 32–38 (2012). 10.1016/j.ejphar.2011.12.042 22266491

[12] Wang, J. et al. Inhibition of aldehyde dehydrogenase 2 by oxidative stress is associated with cardiac dysfunction in diabetic rats. *Mol. Med.* **17**, 172–179 (2011). 20957334 10.2119/molmed.2010.00114 PMC3060979

[13] Zhang, L., Zou, J., Chai, E., Qi, Y. & Zhang, Y. Alpha-lipoic acid attenuates cardiac hypertrophy via downregulation of PARP-2 and subsequent activation of SIRT-1. *Eur. J. Pharm.* **744**, 203–210 (2014). 10.1016/j.ejphar.2014.09.037 25281201

[14] Palikaras, K., Lionaki, E. & Tavernarakis, N. Mechanisms of mitophagy in cellular homeostasis, physiology and pathology. *Nat. Cell Biol.* **20**, 1013–1022 (2018). 30154567 10.1038/s41556-018-0176-2

[15] Munoz, J. P. & Zorzano, A. FUNDC1: a novel protein in cardiac health. *Circulation* **136**, 2267–2270 (2017). 29203567 10.1161/CIRCULATIONAHA.117.031417

[16] Liu, L. et al. Mitochondrial outer-membrane protein FUNDC1 mediates hypoxia-induced mitophagy in mammalian cells. *Nat. Cell Biol.* **14**, 177–185 (2012). 22267086 10.1038/ncb2422

[17] Tang, X. et al. SIRT2 acts as a cardioprotective deacetylase in pathological cardiac hypertrophy. *Circulation* **136**, 2051–2067 (2017). 28947430 10.1161/CIRCULATIONAHA.117.028728 PMC5698109

[18] Liu, J., Killilea, D. W. & Ames, B. N. Age-associated mitochondrial oxidative decay: improvement of carnitine acetyltransferase substrate-binding affinity and activity in brain by feeding old rats acetyl-L-carnitine and/or R-alpha -lipoic acid. *Proc. Natl Acad. Sci. USA* **99**, 1876–1881 (2002). 11854488 10.1073/pnas.261709098 PMC122287

[19] Zhang, W. J. et al. Dietary alpha-lipoic acid supplementation inhibits atherosclerotic lesion development in apolipoprotein E-deficient and apolipoprotein E/low-density lipoprotein receptor-deficient mice. *Circulation* **117**, 421–428 (2008). 18158360 10.1161/CIRCULATIONAHA.107.725275

[20] Yin, L. et al. NR1B2 suppress kidney renal clear cell carcinoma (KIRC) progression by regulation of LATS 1/2-YAP signaling. *J. Exp. Clin. Cancer Res.* **38**, 343 (2019). 31391070 10.1186/s13046-019-1344-3 PMC6686564

[21] Takahashi, T. et al. Cardiac nuclear high-mobility group box 1 ameliorates pathological cardiac hypertrophy by inhibiting DNA damage response. *JACC Basic Transl. Sci.* **4**, 234–247 (2019). 31061925 10.1016/j.jacbts.2018.11.011 PMC6488753

[22] Puchsaka, P., Chaotham, C. & Chanvorachote, P. alpha-Lipoic acid sensitizes lung cancer cells to chemotherapeutic agents and anoikis via integrin beta1/beta3 downregulation. *Int J. Oncol.* **49**, 1445–1456 (2016). 27431988 10.3892/ijo.2016.3624

[23] Ackers-Johnson, M. et al. A simplified, Langendorff-free method for concomitant isolation of viable cardiac myocytes and nonmyocytes from the adult mouse heart. *Circ. Res.* **119**, 909–920 (2016). 27502479 10.1161/CIRCRESAHA.116.309202 PMC5965670

[24] Xu, X., Hua, Y., Nair, S., Bucala, R. & Ren, J. Macrophage migration inhibitory factor deletion exacerbates pressure overload-induced cardiac hypertrophy through mitigating autophagy. *Hypertension* **63**, 490–499 (2014). 24366076 10.1161/HYPERTENSIONAHA.113.02219 PMC3929844

[25] Yin, L. et al. TIP-B1 promotes kidney clear cell carcinoma growth and metastasis via EGFR/AKT signaling. *Aging (Albany, NY)* **11**, 7914–7937 (2019). 31562290 10.18632/aging.102298 PMC6782011

[26] Yin, L. SH3BGRL2 inhibits growth and metastasis in clear cell renal cell carcinoma via activating hippo/TEAD1-Twist1 pathway. *EBioMedicine* **51**, 102596 (2020). 31911271 10.1016/j.ebiom.2019.12.005 PMC7000347

[27] Carey, M. F., Peterson, C. L. & Smale, S. T. Chromatin immunoprecipitation (ChIP). *Cold Spring Harb. Protoc.* **2009**, pdb prot5279 (2009). 20147264 10.1101/pdb.prot5279

[28] Carraway, M. S. et al. Erythropoietin activates mitochondrial biogenesis and couples red cell mass to mitochondrial mass in the heart. *Circ. Res.* **106**, 1722–1730 (2010). 20395592 10.1161/CIRCRESAHA.109.214353 PMC2895561

[29] Marino, A. & Levi, R. Salvaging the ischemic heart: Gi-coupled receptors in mast cells activate a PKCepsilon/ALDH2 pathway providing Anti-RAS cardioprotection. *Curr. Med Chem.* **25**, 4416–4431 (2018). 29446730 10.2174/0929867325666180214115127

[30] Chen, C. H. et al. Activation of aldehyde dehydrogenase-2 reduces ischemic damage to the heart. *Science* **321**, 1493–1495 (2008). 18787169 10.1126/science.1158554 PMC2741612

[31] Yang, M. Y. et al. Activation of aldehyde dehydrogenase 2 slows down the progression of atherosclerosis via attenuation of ER stress and apoptosis in smooth muscle cells. *Acta Pharm. Sin.* **39**, 48–58 (2018). 10.1038/aps.2017.81 PMC5758670 28858301

[32] Dassanayaka, S. et al. Cardiac-specific overexpression of aldehyde dehydrogenase 2 exacerbates cardiac remodeling in response to pressure overload. *Redox Biol.* **17**, 440–449 (2018). 29885625 10.1016/j.redox.2018.05.016 PMC5991908

[33] Jung, J. H. et al. Alpha lipoic acid attenuates radiation-induced thyroid injury in rats. *PLoS ONE* **9**, e112253 (2014). 25401725 10.1371/journal.pone.0112253 PMC4234464

[34] Ryu, S. H. et al. Protective effect of alpha-lipoic acid against radiation-induced fibrosis in mice. *Oncotarget* **7**, 15554–15565 (2016). 26799284 10.18632/oncotarget.6952 PMC4941260

[35] Morsy, M. A., Abdalla, A. M., Mahmoud, A. M., Abdelwahab, S. A. & Mahmoud, M. E. Protective effects of curcumin, alpha-lipoic acid, and N-acetylcysteine against carbon tetrachloride-induced liver fibrosis in rats. *J. Physiol. Biochem.* **68**, 29–35 (2012). 21986891 10.1007/s13105-011-0116-0

[36] Iwata, K. et al. Up-regulation of NOX1/NADPH oxidase following drug-induced myocardial injury promotes cardiac dysfunction and fibrosis. *Free Radic. Biol. Med.* **120**, 277–288 (2018). 29609020 10.1016/j.freeradbiomed.2018.03.053

[37] Krylatov, A. V. et al. Reactive oxygen species as intracellular signaling molecules in the cardiovascular system. *Curr. Cardiol. Rev.* **14**, 290–300 (2018). 29962348 10.2174/1573403X14666180702152436 PMC6300799

[38] Oka, T. et al. Mitochondrial DNA that escapes from autophagy causes inflammation and heart failure. *Nature* **485**, 251–255 (2012). 22535248 10.1038/nature10992 PMC3378041

[39] Saito, T. et al. An alternative mitophagy pathway mediated by Rab9 protects the heart against ischemia. *J. Clin. Investig.* **129**, 802–819 (2019). 30511961 10.1172/JCI122035 PMC6355232

[40] Wang, B. et al. AMPKalpha2 protects against the development of heart failure by enhancing mitophagy via PINK1 phosphorylation. *Circ. Res.* **122**, 712–729 (2018). 29284690 10.1161/CIRCRESAHA.117.312317 PMC5834386

[41] Zhou, H. et al. Ripk3 induces mitochondrial apoptosis via inhibition of FUNDC1 mitophagy in cardiac IR injury. *Redox Biol.* **13**, 498–507 (2017). 28732308 10.1016/j.redox.2017.07.007 PMC5828768

[42] Zhou, H. et al. Pathogenesis of cardiac ischemia reperfusion injury is associated with CK2alpha-disturbed mitochondrial homeostasis via suppression of FUNDC1-related mitophagy. *Cell Death Differ.* **25**, 1080–1093 (2018). 29540794 10.1038/s41418-018-0086-7 PMC5988750

[43] Ji, W. et al. Aldehyde dehydrogenase 2 has cardioprotective effects on myocardial ischaemia/reperfusion injury via suppressing mitophagy. *Front. Pharm.* **7**, 101 (2016). 10.3389/fphar.2016.00101 PMC4838626 27148058

[44] Lefebvre, V. et al. Genome-wide RNAi screen identifies ATPase inhibitory factor 1 (ATPIF1) as essential for PARK2 recruitment and mitophagy. *Autophagy* **9**, 1770–1779 (2013). 24005319 10.4161/auto.25413

[45] He, C. et al. Beclin 2 functions in autophagy, degradation of G protein-coupled receptors, and metabolism. *Cell* **154**, 1085–1099 (2013). 23954414 10.1016/j.cell.2013.07.035 PMC4231430

[46] Chen, S., Liu, G., Long, M., Zou, H. & Cui, H. Alpha lipoic acid attenuates cadmium-induced nephrotoxicity via the mitochondrial apoptotic pathways in rat. *J. Inorg. Biochem.* **184**, 19–26 (2018). 29654931 10.1016/j.jinorgbio.2018.04.001

[47] Jin, S. et al. ALDH2(E487K) mutation increases protein turnover and promotes murine hepatocarcinogenesis. *Proc. Natl Acad. Sci. USA* **112**, 9088–9093 (2015). 26150517 10.1073/pnas.1510757112 PMC4517197

