# Supplementary material for: Alpha-lipoic acid protects against pressure overload-induced heart failure via ALDH2-dependent Nrf1-FUNDC1 signaling
Source: Cell Death Dis. 2020 Jul 30;11(7):599. doi: 10.1038/s41419-020-02805-2 (PMC7393127; doi:10.1038/s41419-020-02805-2)
Supplement: Supplementary file 1 — Supplementary Information [file 41419_2020_2805_MOESM1_ESM.docx]

**Supplementary Figure legend**

**Figure I.** (A) Left ventricular end-systolic diameter (LVESD, mm); Left ventricular end-diastolic diameter (LVEDD, mm) and Left ventricular diastole posterior wall dimension (LVPWD, mm) in WT mice (n=8-10 mice per group); (B) Hypertrophic genes expression (Bnp, Anp, β-Mhc) and fibrotic genes expression (Ctgf, Col1a, Col3a) in WT mice (n=6 mice per group); (C) mtDNA copy number after sham or TAC operation with or without α-LA treatment quantified by real-time PCR (n=6 mice per group); (D) Western blots of SOD2 and β-actin (loading control); (E) Detailed ALDH2 gene expression from heatmap of different groups in mRNA array (n=4 mice per group); (F) Immunohistochemistry staining of FUNDC1 (Down: scale bar=50 μm). Mean ± SEM, *P<0.05, **P<0.01, ***P<0.001, ****P<0.0001. Statistical analysis was carried out by a one-way ANOVA analysis followed by Tukey’s test for post hoc analysis.

**Figure II.** (A) Change of DNA and protein level in ALDH2^-/-^ mice. (B) Left ventricular end-systolic diameter (LVESD, mm); Left ventricular end-diastolic diameter (LVEDD, mm) and Left ventricular diastole posterior wall dimension (LVPWD, mm) in WT and ALDH2^-/-^ mice (n=8-20 mice per group); (C) Quantitative analysis of cardiomyocyte cross-sectional areas (CSA) (n=6-9 mice per group); (D) Quantitative analysis of myocardial fibrosis (n=6 mice per group); (E) Hypertrophic genes expression (Bnp, Anp, β-Mhc) and fibrotic genes expression (Ctgf, Col1a, Col3a) in WT and ALDH2^-/-^ mice (n=6 mice per group); (F-G) Terminal dexynucleotidyl transferase (TdT)-mediated dUTP nick end labeling staining (TUNEL, 400X, scale bar=20 μm); (H) Quantitative analysis of myocardial apoptosis (n=6 mice per group); (I) Dihydroethidium (DHE, 200X, scale bar=50 μm) staining; (J) Quantitative analysis of myocardial ROS levels (n=6 mice per group); (K) Western blots of FUNDC1, LC3, P62, and β-actin (loading control) between ALDH2^-/-^- TAC + Control and ALDH2^-/-^- TAC + α-LA group; (L-N) Quantitative analysis of expressions of FUNDC1/β-actin, LC3II/LC3I, P62/β-actin between ALDH2^-/-^- TAC + Control and ALDH2^-/-^- TAC + α-LA group (n=6 mice per group). Mean ± SEM, *P<0.05, **P<0.01, ***P<0.001, ****P<0.0001. Statistical analysis was carried out by a one-way ANOVA analysis followed by Tukey’s test for post hoc analysis or two-tailed Student’s t-test.

**Figure III.** (A) Knock down ALDH2 by pLKO/pLKO.1-ALDH2 in NRCMs cells and chose the higher efficiency sh1-ALDH2 for further investigation; (B-E) Quantitative analysis of expressions of Nrf1/β-actin, Nrf2/β-actin, PFKP/β-actin and LDHA/β-actin (n=6 samples per group); (F) Overexpression of ALDH2 by pWPI/pWPI-ALDH2 in NRCMs cells; (G) Knock down Nrf1 by pLKO/pLKO.1-Nrf1 in NRCMs cells and chose the higher efficiency sh1-Nrf1 for further investigation; (H) Quantitative analysis of expressions of ALDH2/β-actin, FUNDC1/β-actin and Nrf1/β-actin in NRCMs after transfection (n=6 samples per group). Mean ± SEM, *P<0.05, **P<0.01, ***P<0.001, ****P<0.0001. Statistical analysis was carried out by a one-way ANOVA analysis followed by Tukey’s test for post hoc analysis.

**Supplementary Table legend**

**Table S I.** The mouse mitophagy PCR array

**Table S II.** The primer sequences

**Table S III.** The sequences of oligonucleotides

**Table S VI**. Three putative Nrf1REs within the proximal 2kb promoter region
