# Supplementary figures and images for: Alpha-lipoic acid protects against pressure overload-induced heart failure via ALDH2-dependent Nrf1-FUNDC1 signaling
Source: Cell Death Dis. 2020 Jul 30;11(7):599. doi: 10.1038/s41419-020-02805-2 (PMC7393127; doi:10.1038/s41419-020-02805-2)

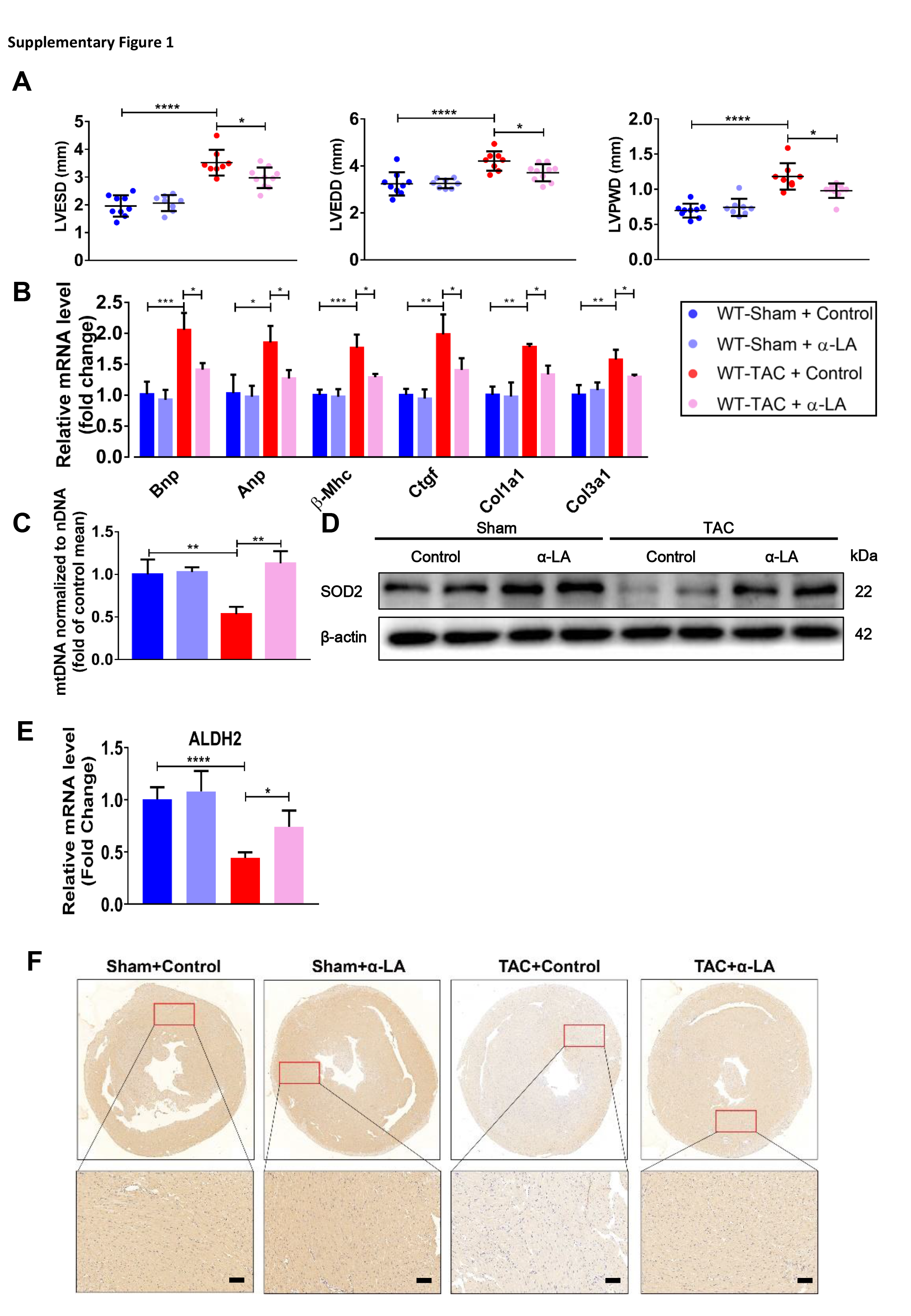

Supplement: Supplementary file 2 — Supplementary Figure 1 [file 41419_2020_2805_MOESM2_ESM.tif]

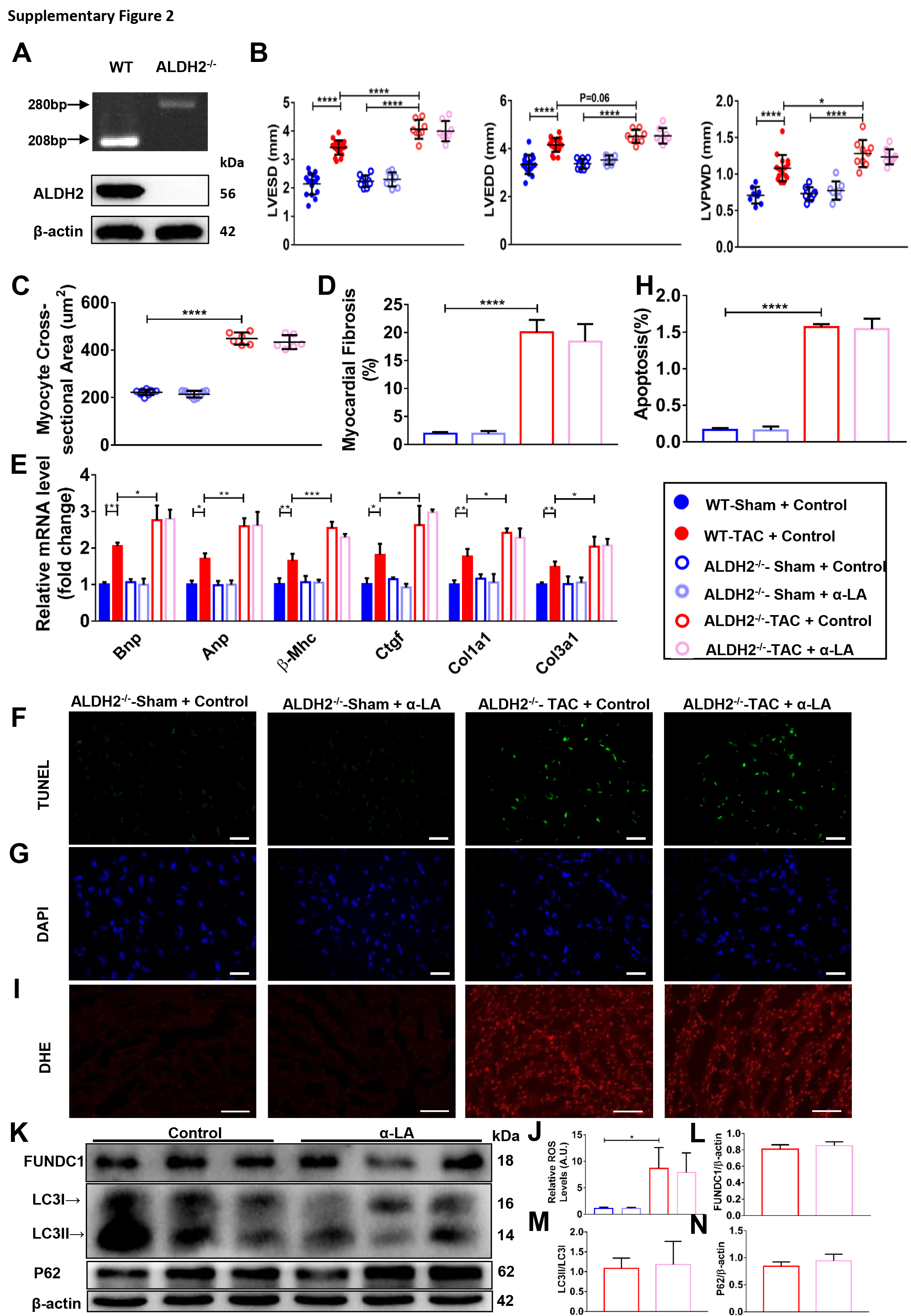

Supplement: Supplementary file 3 — Supplementary Figure 2 [file 41419_2020_2805_MOESM3_ESM.tif]

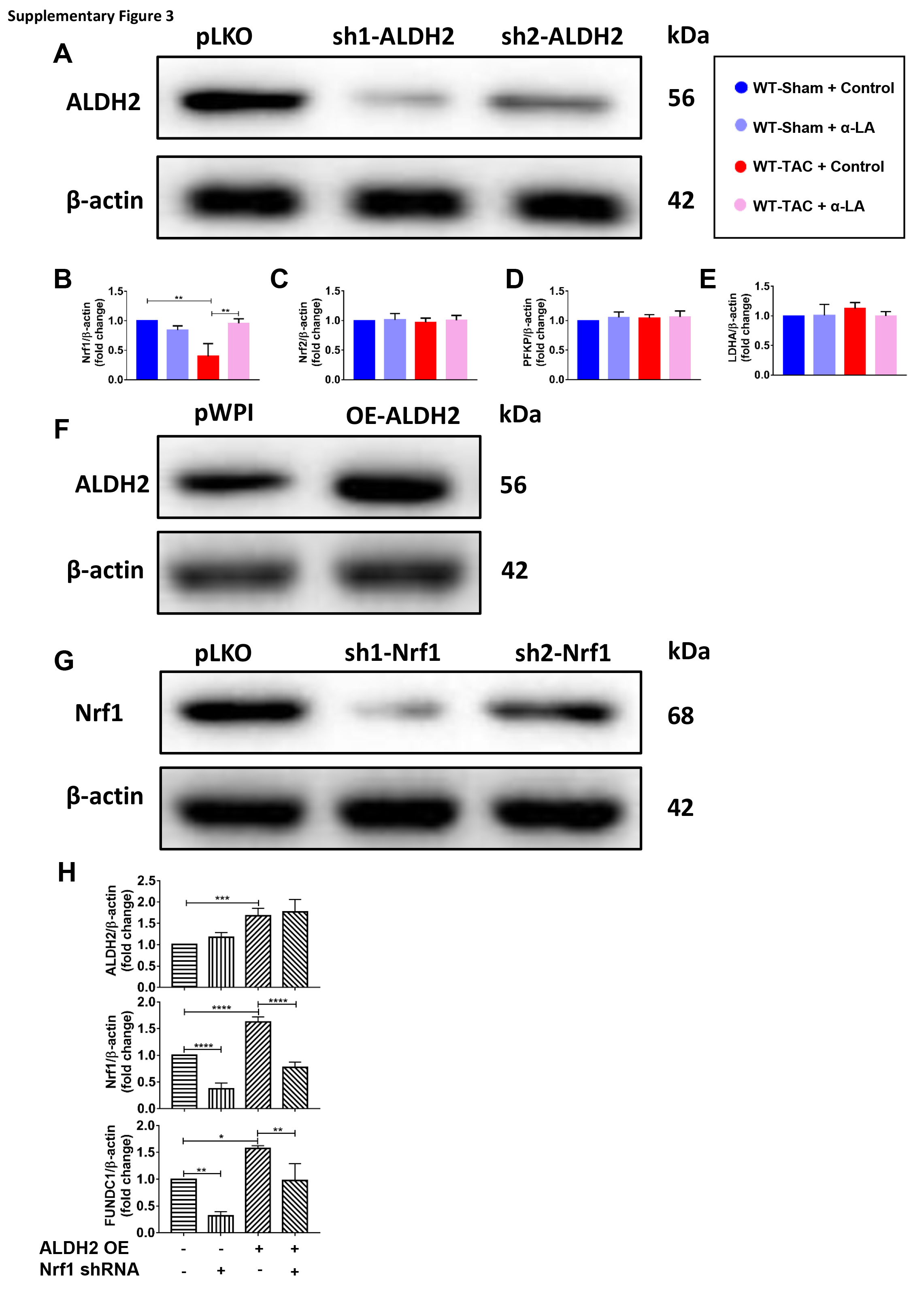

Supplement: Supplementary file 4 — Supplementary Figure 3 [file 41419_2020_2805_MOESM4_ESM.tif]
